# Homeodomain Transcription Factor Meis1 Is a Critical Regulator of Adult Bone Marrow Hematopoiesis

**DOI:** 10.1371/journal.pone.0087646

**Published:** 2014-02-03

**Authors:** Reina Ariki, Satoru Morikawa, Yo Mabuchi, Sadafumi Suzuki, Mayuka Nakatake, Kentaro Yoshioka, Shinya Hidano, Hiromitsu Nakauchi, Yumi Matsuzaki, Takuro Nakamura, Ryo Goitsuka

**Affiliations:** 1 Division of Development and Aging, Research Institute for Biomedical Sciences, Tokyo University of Science, Noda, Chiba, Japan; 2 Department of Physiology, Keio University School of Medicine, Shinjuku-ku, Tokyo, Japan; 3 Division of Carcinogenesis, The Cancer Institute, Japanese Foundation for Cancer Research, Koto-ku, Tokyo, Japan; 4 Center for Stem Cell Biology and Regenerative Medicine, The Institute of Medical Science, The University of Tokyo, Minato-ku, Tokyo, Japan; Emory University, United States of America

## Abstract

Hematopoietic stem cells in the bone marrow have the capacity to both self-renew and to generate all cells of the hematopoietic system. The balance of these two activities is controlled by hematopoietic stem cell-intrinsic regulatory mechanisms as well as extrinsic signals from the microenvironment. Here we demonstrate that Meis1, a TALE family homeodomain transcription factor involved in numerous embryonic developmental processes, is selectively expressed in hematopoietic stem/progenitor cells. Conditional Meis1 knockout in adult hematopoietic cells resulted in a significant reduction in the hematopoietic stem/progenitor cells. Suppression of hematopoiesis by *Meis1* deletion appears to be caused by impaired self-renewal activity and reduced cellular quiescence of hematopoietic stem/progenitor cells in a cell autonomous manner, resulting in stem cell exhaustion and defective long-term hematopoiesis. Meis1 deficiency down-regulated a subset of Pbx1-dependent hematopoietic stem cell signature genes, suggesting a functional link between them in the maintenance of hematopoietic stem/progenitor cells. These results show the importance of Meis1 in adult hematopoiesis.

## Introduction

Hematopoiesis in adult animals is sustained by a small population of multipotent hematopoietic stem cells (HSCs), which maintain the capacity for both self-renew and differentiation, thereby generating all the cell types of the hematopoietic system. In normal mice and humans, HSCs are localized predominantly in a specialized microenvironment (niche) within the bone marrow (BM), where signals from cells in the surrounding niche maintain them in a state of slow cell cycling or quiescence [1]–[3]. The self-renewal of postnatal HSCs is closely coupled with this slow cell cycling or quiescence and is a critical requirement for long-term maintenance of the self-renewing HSC compartment.

HSC quiescence is controlled by both HSC-intrinsic mechanisms and extrinsic factors from the BM microenvironment [1]. Several transcription factors have been implicated in the regulation of HSC quiescence, including Gfi-1, Pbx1 and MEF/ELF4 [4]–[7]. With regard to HSC-extrinsic niche-derived factors, it has been reported that angiopoietin-1 and thrombopoietin regulate the quiescence of HSCs in the BM through receptors expressed on HSCs [8]–[10]. Furthermore, hypoxia inducible factor-1α (HIF-1α), a transcription factor that is transcribed and stabilized under low oxygen conditions such as in the BM niche for HSCs, has been shown to regulate HSC quiescence as well as metabolism [11], [12]. Thus it is an important molecular link between extrinsic and intrinsic regulatory mechanisms modulating HSC quiescence.

The *Meis1* gene encodes a TALE-family transcription factor that was first identified as a common retroviral integration site in BXH2 murine myeloid leukemia [13], [14]. Meis1 functions as a DNA-binding cofactor of Hox proteins through interaction with Pbx, a member of another TALE homeodomain subfamily of transcription factors [15]. Meis1 by itself does not transform hematopoietic cells. However, it cooperates with Hoxa9 to significantly accelerate Hox-induced leukemogenesis [16]. Moreover, *Meis1* as well as *Hoxa9* have been shown to be the most critical downstream targets of *Mixed Lineage Leukemia* (*MLL*) fusion proteins [17], and their co-expression is sufficient to induce acute myeloid leukemia [14], [16], recapitulating *MLL-ENL*-induced immortalization of myeloid progenitor cells [18]. In addition, Meis1 regulates the differentiation arrest, cycling activity and self-renewal of *MLL* leukemia cells, a critical rate-limiting determinant for establishing leukemia stem cell potential [19].

In contrast to the established role of Meis1 in leukemia development, its function in postnatal hematopoiesis, especially in HSCs as well as hematopoietic progenitor cells (HPCs), remains uncertain. Targeted *Meis1* homozygous deletion in mice results in lethality by embryonic day 14.5 with hematopoietic and vascular defects [20], [21]. In *Meis1*-deficient fetal liver, HSC compartments were severely affected and colony formation potential as well as the capacity to repopulate lethally irradiated recipient mice were profoundly impaired, suggesting a critical role of Meis1 in HSC/HPC maintenance. Furthermore, Meis1 is required for transcriptional activation of *Hif1a* in HSCs through binding to its conserved consensus sequence within the first intron of *Hif1a* [11]. Thus, these observations support the hypothesis that Meis1 has a critical role in the regulation of HSC/HPC maintenance. However, a comprehensive analysis of Meis1 function has been hampered because of the embryonic lethality of the *Meis1* mutation.

In the present study, we employed a genetic approach to conditionally inactivate *Meis1* in the mouse hematopoietic system *in vivo*. Our current analysis reveals that Meis1 is a critical regulator of hematopoiesis in the adult BM.

## Results

### Impaired hematopoiesis in the absence of Meis1

As predicted from a gene expression database search [22], *Meis1* was highly expressed in both CD34^−^ and CD34^+^Lin^−^Sca-1^+^c-Kit^+^ (LSK) cells, whereas its expression became undetectable in most of the lineage-committed hematopoietic cells (**Figure S1**). *Meis2* and *Meis3* transcripts were undetectable in any of the hematopoietic lineage cells tested, therefore Meis1 is the sole Meis transcription factor family member expressed in hematopoietic cells under physiological conditions.

The early embryonic lethality resulting from germ-line deletion of the *Meis1* gene precludes any study of postnatal hematopoiesis in the BM. Therefore, we generated mice harboring conditional alleles of *Meis1* (*Meis1^fl^*), in which *Meis1* exon 8 encoding the homeodomain was flanked by *lox*P sites (**Figure S2A and B**). The *Meis1^fl/fl^* mice were born normally and appeared healthy. Given the expression pattern of *Meis1*, we chose to study the consequence of Meis1 ablation in the HSC/HPC by crossing the *Meis1^fl^* conditional-knockout strain with the interferon-responsive *Mx1-Cre* transgenic line, which achieves highly efficient excision of *lox*P-flanked DNA in hematopoietic cells *in vivo* after induction with poly(I:C) [23]. As shown in **Figure S2C**, four intraperitoneal injections of poly(I:C) into *Mx1-Cre^+^ Meis1^fl/fl^* mice was sufficient to induce complete deletion of *Meis1* exon 8 in BM cells. Since both *Mx1-Cre^+^ Meis1* ^fl/+^ and *Meis1^fl/fl^* mice displayed similar phenotypes upon poly(I:C) treatment (data not shown), we used *Meis1^fl/fl^* mice as controls unless otherwise indicated.

**Figure 1:**
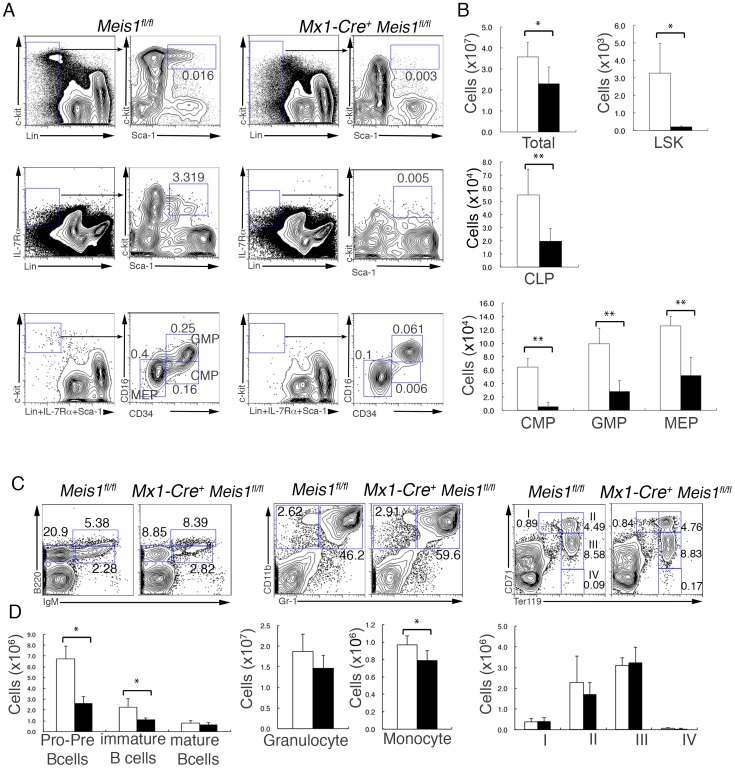
Loss of Meis1 leads to a depletion of hematopoietic progenitor cells from the bone marrow. (A) Representative flow cytometric profiles of hematopoietic progenitor cell populations from *Mx1-Cre* ^+^ *Meis1* ^fl/fl^ and control *Meis1* ^fl/fl^ mice three weeks after poly(I:C) treatment. Gates used to identify progenitor populations are outlined, and rightward arrows within the plots indicate their relationship to subsequent plots showing the progenitor populations. Numbers adjacent to outlined areas indicate percentage of gated cells in total BM mononuclear cells. (B) Absolute numbers of the indicated cell populations per two femurs in poly(I:C)–treated *Mx1-Cre* ^+^ *Meis1* ^fl/fl^ (solid bars) and control *Meis1* ^fl/fl^ (open bars) mice (mean and SD; n = 4). (C) Representative flow cytometric profiles of lineage-committed cell populations. (D) Absolute numbers of the indicated cell populations per two femurs in poly(I:C)–treated *Mx1-Cre* ^+^ *Meis1* ^fl/fl^ (solid bars) and control *Meis1* ^fl/fl^ (open bars) mice (mean and SD; n = 4). Pro- and pre-B cells (B220^low^ IgM^−^), immature B cells (B220^low^ IgM^+^), mature B cells (B220^+^ IgM^+^), granulocytes (Gr-1^+^CD11b^+^), monocytes (Gr-1^−^ CD11b^+^), proerythroblasts (I; Ter119^low^ CD71^high^), basophilic erythroblast (II; Ter119^high^ CD71^high^) and late erythroblasts (III; Ter119^high^ CD71^int^ and IV; Ter119^high^ CD71^low^). *p<0.05 and **p<0.01.

We analyzed hematopoiesis in adult *Mx1-Cre^+^ Meis1^fl/fl^* mice three weeks after poly(I:C) treatment compared to similarly treated *Meis1^fl/fl^* littermates. Three weeks after induced deletion of *Meis1*, the total number of BM cells was slightly but significantly reduced in *Mx1-Cre^+^ Meis1^fl/fl^* mice (**Figure 1A and B**). At the HSC/HPC level, LSK cells were almost undetectable in *Mx1-Cre^+^ Meis1^fl/fl^* mice (**Figure 1A and B**). The relative proportion and the total number of Lin^−^IL-7R^+^Sca-1^int^c-Kit^+^ (CLP) cells was significantly lower in *Mx1-Cre^+^ Meis1^fl/fl^* mice than in control *Meis1^fl/fl^* mice. In addition, the Lin^−^Sca-1^−^c-Kit^+^FcγRII/III^int^ CD34^high^ (CMP) population was nearly absent, and the cell populations at the subsequent developmental stages, Lin^−^Sca-1^−^c-Kit^+^FcγRII/III^high^ CD34^high^ (GMP) as well as Lin^−^Sca-1^−^c-Kit^+^FcγRII/III^−^ CD34^high^ (MEP), were also significantly reduced in *Mx1-Cre^+^ Meis1^fl/fl^* mice compared to control *Meis1^fl/fl^* mice (**Figure 1A and B**).

In contrast to the profound decrease in lineage-negative hematopoietic precursors, the frequencies and the total numbers of granulocytes (Gr-1^+^CD11b^+^) was unaffected in *Mx1-Cre^+^ Meis1^fl/fl^* mice and the number of monocytes (Gr-1^−^ CD11b^+^) was only slightly reduced (**Figure 1C and D**), in spite of the efficient deletion of the floxed *Meis1* alleles in most of these cells (**Figure S3**). The differentiation profile of erythroid lineage cells from proerythroblast (I; Ter119^low^ CD71^high^), basophilic erythroblast (II; Ter119^high^ CD71^high^), to late erythroblasts (III, Ter119^high^ CD71^int^, and IV, Ter119^high^ CD71^low^) was also intact in *Mx1-Cre^+^ Meis1^fl/fl^* mice. (**Figure 1C and D**). Furthermore, consistent with the essential role of Meis1 in fetal megakaryopoiesis [20], [21], the proportion and number of Lin^−^ cKit^+^ CD41^+^ megakaryocyte precursors in the BM, which contain CFU-Meg [24], was also significantly reduced in *Mx1-Cre^+^ Meis1^fl/fl^* mice; however, at this time point after Meis1 deletion, the number of c-Kit^−^ CD41^+^ mature megakaryocytes was unaffected in *Mx1-Cre^+^ Meis1^fl/fl^* mice (**Figure S4**), as observed in other lineage-committed cells. The proportions and the total numbers of early B-lineage cells containing pro- and pre-B cell populations (B220^low^ IgM^−^) as well as immature B cells (B220^low^ IgM^+^) in the BM were also significantly reduced in *Mx1-Cre^+^ Meis1^fl/fl^* mice (**Figure 1C and D**), which is consistent with the relatively low expression of *Meis1* in bone marrow B220^+^ B-lineage cells (**Figure S1**). T cell numbers in the thymus were also significantly reduced in *Mx1-Cre^+^ Meis1^fl/fl^* mice compared to those in the control mice (**Figure S5**). These results demonstrated that acute *Meis1* loss results in severe defects in early hematopoiesis at the progenitor levels rather than in late lineage-committed cells.

### Defects in the HSC compartment in the absence of Meis1

**Figure 2:**
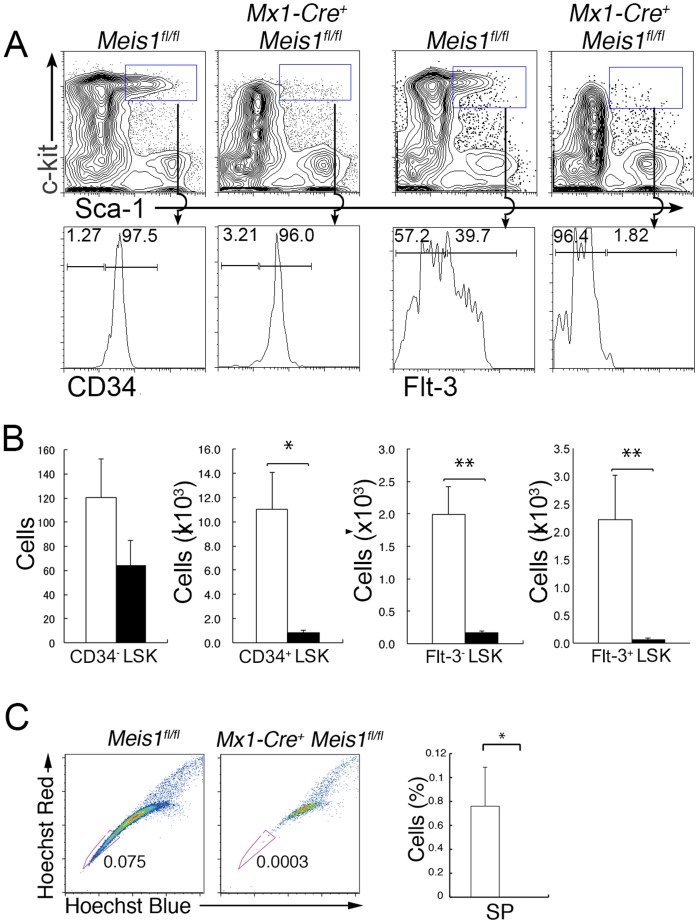
Loss of Meis1 causes profound defects in the HSC compartment. (A) Representative flow cytometric profiles of LSK cells in BM depleted of lineage-positive cells from *Mx1-Cre* ^+^ *Meis1* ^fl/fl^ and control *Meis1* ^fl/fl^ mice three weeks after poly(I:C) treatment. Gates used to identify LSK cell populations are outlined, and downward arrows indicate their relationship to subsequent histograms showing the expression of CD34 or Flt3 in these cell populations. Numbers in the histograms indicate percent events in each gate. (B) Absolute numbers of the indicated LSK cell populations per two femurs from poly(I:C)–treated *Mx1-Cre^+^ Meis1* ^fl/fl^ (solid bars) and control *Meis1* ^fl/fl^ (open bars) mice (mean and SD; n = 4). *p<0.05 and **p<0.01. (C) Representative flow cytometric profiles of side population (SP) cells within the LSK population from *Mx1-Cre* ^+^ *Meis1* ^fl/fl^ and control mice one week after poly(I:C) treatment. Bar graphs shown on the right represent the percentage of SP cells in the LSK cell population from poly(I:C)–treated *Mx1-Cre* ^+^ *Meis1* ^fl/fl^ (solid bars) and control *Meis1* ^fl/fl^ (open bars) mice (mean and SD; n = 3). *p<0.005.

The LSK population is heterogeneous and contains both HSCs and HPCs with multi-lineage potential but limited self-renewal capacity [25], [26]. Therefore, we examined the LSK compartment in detail by staining for CD34 or Flt3 expression. The total number of CD34^−^ LSK cells was lower in *Mx1-Cre^+^ Meis1^fl/fl^* mice than in control *Meis1^fl/fl^* mice (**Figure 2A and B**), and the defect was more profound in CD34^+^ LSK cells as well as in Flt3^−^ and Flt3^+^ LSK cells (**Figure 2A and B**). CD34^+^ LSK cells as well as Flt3^+^ LSK cells were about 20-fold and 100-fold lower in *Mx1-Cre^+^ Meis1^fl/fl^* mice than in control *Mx1-Cre^−^ Meis1^fl/fl^* mice, respectively (**Figure 2A and B**). Furthermore, “side population” (SP) cells, which represent quiescent HSCs [27], but not non-SP cells, in the LSK population were almost completely missing from *Mx1-Cre^+^ Meis1^fl/fl^* mice upon induction of *Meis1* deletion (**Figure 2C**). These results suggest that the stages among LSK cells that require Meis1 appear to be HSCs.

### Meis1 regulates self-renewal of HSC in a cell autonomous manner

**Figure 3:**
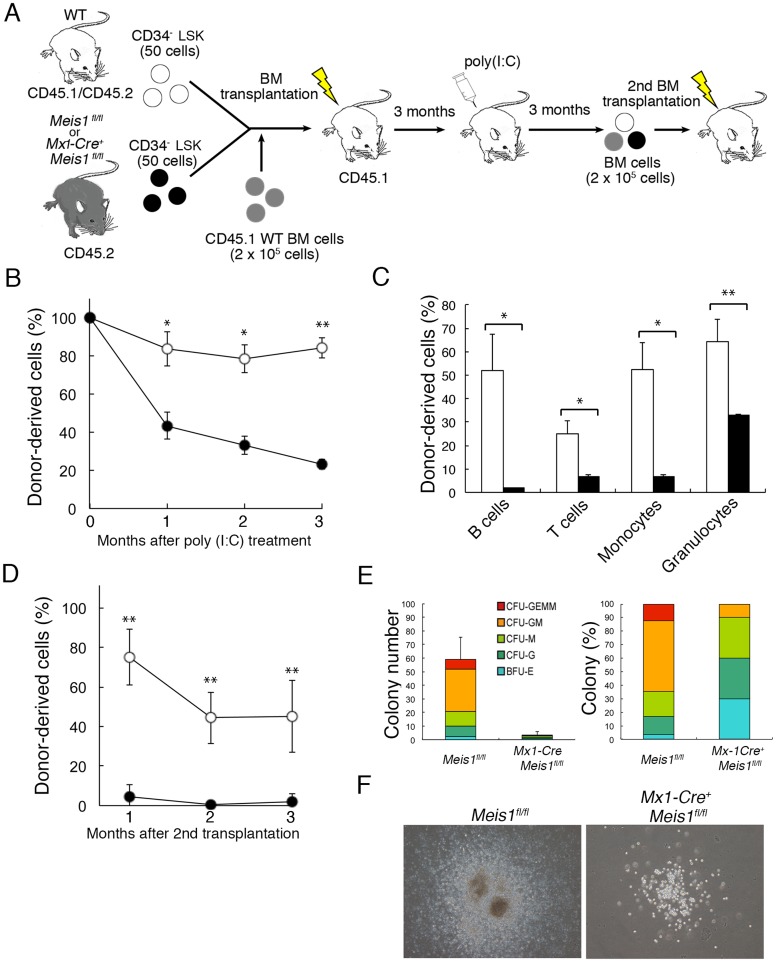
Meis1 regulates self-renewal of HSCs in a cell autonomous manner. (A) Experimental strategy for analyzing the function of Meis1 in HSCs. Mice with chimeric BM were generated by transplanting CD34^−^ LSK cells (50 cells/mouse) from *Mx1-Cre* ^+^ *Meis1* ^fl/fl^ or control *Meis1* ^fl/fl^ mice (CD45.2) and an equal number of CD34^−^ LSK cells from wild-type mice (CD45.1/CD45.2) with CD45.1 BM supports cells in lethally irradiated CD45.1 recipient mice. A subset of mice was treated with poly(I:C) three months after transplantation. (B) Mean percentages of CD45.2^+^ cells ± SD in the peripheral blood derived from *Meis1* ^fl/fl^ (n = 6; open circles) and *Mx1-Cre^+^ Meis1* ^fl/fl^ (n = 6; closed circles) CD34^−^ LSK cells after poly(I:C) treatment. Initial engraftment of CD45.2^+^ was normalized to 100% for each mouse. *p<0.05 and **p<0.01. (C) Histogram represents mean (± SD) contribution of the indicated splenic cell populations derived from *Meis1* ^fl/fl^ (n = 6; open bars) and *Mx1-Cre* ^+^ *Meis1* ^fl/fl^ (n = 6; solid bars) CD34^−^ LSK cells three months after poly(I:C) treatment. *p<0.05 and **p<0.01. (D) Mean percentages of CD45.2^+^ cells ± SD in the peripheral blood derived from *Meis1* ^fl/fl^ (n = 6; open circles) or *Mx1-Cre^+^ Meis1* ^fl/fl^ (n = 6; closed circles) CD34^−^ LSK cells after secondary transplantation. *p<0.05 and **p<0.01. (E) Total numbers (left) and proportions (right) of different colony types produced by CD34^−^ LSK cells from poly(I:C)-treated *Mx1-Cre^+^ Meis1* ^fl/fl^ and control *Meis1* ^fl/fl^ mice (n = 3 per each group). Cultures were assessed on day 14 for granulocyte (CFU-G), monocytes (CFU-M), granulocyte-monocyte (CFU-GM), erythroid (BFU-E) and mixed (CFU-GEMM) colony formation. The data are the means of three-independent experiments. (F) Representative photographs of 14 day colonies derived from CD34^−^ LSK cells from poly(I:C)-treated *Mx1-Cre^+^ Meis1* ^fl/fl^- and control *Meis1* ^fl/fl^ mice (×40 magnification).

Although the above data suggested that *Meis1* deficiency caused a loss of HSC/HPCs, *Mx1-Cre*-mediated induction of *Meis1* loss is not strictly limited to hematopoietic cells [23]. Thus, to determine whether the *Meis1*-deficient phenotype is HSC autonomous or dependent on the lack of Meis1 in the HSC niche, we induced the loss of Meis1 in pre-established BM chimeric mice. Irradiated recipient mice (CD45.1^+^) were transplanted with a 1∶1 mixture of donor CD34^−^ LSK cells from uninduced *Mx1-Cre^+^ Meis1^fl/fl^* mice or control *Meis1^fl/fl^* mice (CD45.2^+^) and competitor CD34^−^ LSK cells from wild-type mice (CD45.1/CD45.2). Three months later, chimeric mice were injected with poly(I:C) to induce *Meis1* loss, and the fraction of donor-derived peripheral blood leukocytes (PBL) was subsequently monitored (**Figure 3A**). In contrast to untreated recipient mice, the poly(I:C)-injected recipients manifested a sharp decline of *Mx1-Cre^+^ Meis1^fl/fl^* cell-derived PBL, as compared to control *Meis1^fl/fl^* cell-derived PBL (**Figure 3B**). Three months after induction of *Meis1* deletion, a significant reduction of *Meis1*-deficient donor cells of all hematopoietic cell types, including B cells, T cells, monocytes and granulocytes in the spleen was confirmed (**Figure 3C**). Furthermore, *Meis1*-deficient donor cells were undetectable from one to three months after the secondary transplantation of BM cells from these primary recipient chimeric mice into irradiated secondary recipients (**Figure 3D**). These data indicate that Meis1 regulates the long-term maintenance of HSC/HPCs in a cell autonomous manner.

We next examined the impact of *Meis1* loss on HSC/HPC proliferation and differentiation using *in vitro* colony forming assays. As shown in **Figure 3E**, *Mx1-Cre^+^ Meis1^fl/fl^* CD34^−^ LSK cells generated ten-fold fewer colonies compared to control *Meis1^fl/fl^* CD34^−^ LSK cells. Furthermore, colonies derived from *Mx1-Cre^+^ Meis1^fl/f l^* cells were significantly smaller in size than those from the control cells, indicating that *Meis1*-deficient cells had lost replication potential (**Figure 3F**). Despite the significant decrease in the number and size of colonies derived from *Meis1*-deficient cells, they still retained the potential to give rise to CFU-GM, CFU-M, CFU-G and BFU-E, but failed to form CFU-GEMM (**Figure 3E** **, right**). These results suggest that the hematopoietic abnormalities arising from *Meis1*-deficiency result from the defective self-renewal capacity of HSCs.

### Meis1 regulates the cell cycle status of HSC/HPCs

**Figure 4:**
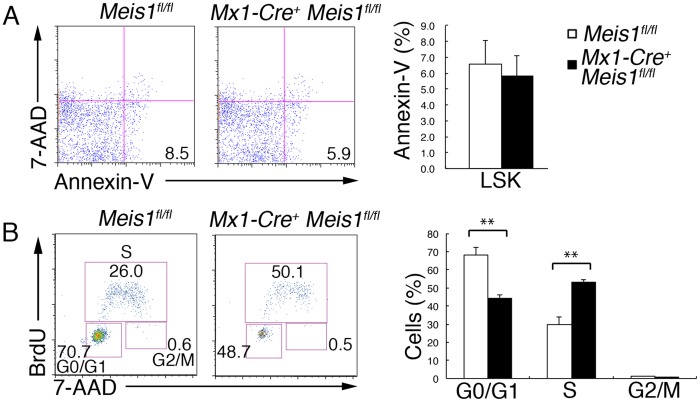
Meis1 regulates cell cycle of the HSC compartment. (A) Representative flow cytometric profiles showing Annexin V and 7-AAD staining of LSK cells from *Mx1-Cre^+^ Meis1* ^fl/fl^ and control mice one week after poly(I:C) treatment. Bar graphs on the right represent the percentages of apoptotic LSK cells (anexin V^+^ 7-AAD^−^) cells from poly(I:C)–treated *Mx1-Cre* ^+^ *Meis1* ^fl/fl^ (solid bars) and control *Meis1* ^fl/fl^ (open bars) mice (mean and SD; n = 3). (B) Representative flow cytometric profiles showing BrdU incorporation and 7-AAD staining of LSK cells from *Mx1-Cre Meis1* ^fl/fl^ and control mice one week after poly(I:C) treatment. Bar graphs shown on the right represent the percentages of cells in G0/G1-, S- and G2/M-phase of the cell cycle in the LSK cell population from poly(I:C)–treated *Mx1-Cre* ^+^ *Meis1* ^fl/fl^ (solid bars) and control *Meis1* ^fl/fl^ (open bars) mice (mean and SD; n = 3). **p<0.01.

To understand the mechanism of HSC/HPC exhaustion in the absence of Meis1, we assessed apoptosis of HSCs using Annexin V staining of LSK cells at an early time point (one week) after deletion of *Meis1*, when no decrease in cell numbers or increase in apoptosis of any hematopoietic cell compartments, including LSK cells and Lin^−^ Sca1^−^ c-Kit^+^ (LK) cells, was detectable (data not shown). As shown in **Figure 4A**, we found no difference in the proportion of Annexin V^+^ LSK cells between *Mx1-Cre^+^ Meis1^fl/fl^* and control *Meis1^fl/fl^* mice. We next examined cell cycle status of HSC/HPCs because it is important for maintenance of their long-term self-renewal capacity [28]. Cells that had entered S phase, as detected by BrdU incorporation, were markedly increased in *Mx1-Cre^+^ Meis1^fl/fl^* LSK cells compared with control *Meis1^fl/fl^* LSK cells, and this was accompanied by a significant reduction in the fraction of *Mx1-Cre^+^ Meis1^fl/fl^* LSK cells in G0/G1 (**Figure 4B**). Taken together, these data suggest that Meis1 regulates the cell cycle of HSC/HPCs, and its absence might lead to the exhaustion of HSC/HPCs.

### Meis1 regulates expression of genes involved in HSC/HPC maintenance

**Figure 5:**
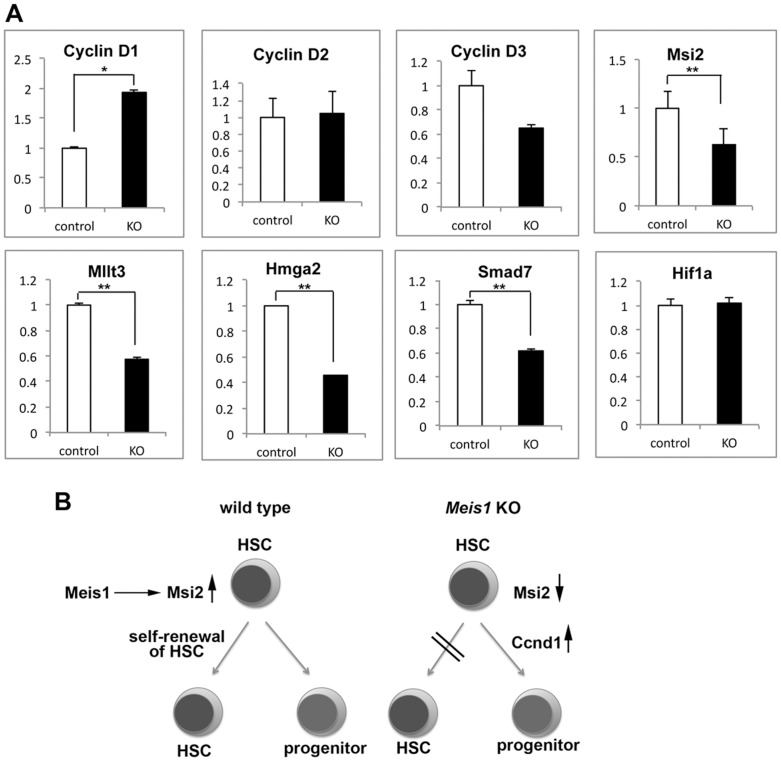
Meis1 controls expression of genes involved in HSC cell cycle and maintenance. (A) Alterations of gene expression in LSK cells induced by *Meis1* loss were analyzed by quantitative RT-PCR. Histograms show the indicated transcripts in sorted LSK cells from *Mx1-Cre^+^ Meis1* ^fl/fl^ mice (open bars) and control *Meis1* ^fl/fl^ mice (solid bars) one week post poly(I:C) treatment. Data were normalized to *Gapdh* expression and the level of each transcript in LSK cells from control mice was arbitrarily set to 1. Data are the means and standard deviations of three independent experiments. *p<0.05 and **p<0.01. (B) Models illustrating potential mechanisms of Meis1 function in the maintenance of HSC.

Finally, we examined the impact of the loss of Meis1 on gene expression in LSK cells. An initial screening by global gene profiling of LSK cells from control and *Mx1-Cre^+^ Meis1^fl/fl^* mice one week following poly(I:C) treatment revealed a profound reduction in the expression of several genes in Meis1-deficient LSK cells, including *Egr2*, *Trib2*, *Hmga2*, *Hlf*, *Mllt3*, *Smad7*, *Nfatc2*, *Skil*, and *Msi2* (**Table S1**). These genes are also affected by Pbx1-deficiency in HSCs [5]. The change in expression of several genes was verified by quantitative PCR (**Figure 5A**). Although *Hif1a* was reported to be a direct transcriptional target of Meis1 in HSCs [11], its expression was not significantly affected upon *Meis1* deletion in LSK cells. However, the expression of *Flt3* in Meis1-deficient LSK cells was reduced by about eight-fold, compared to that in controls (**Table S1**), an observation that is consistent with a previous observation that *Flt3* is a direct transcriptional target of Meis1 [29]. We also quantified the expression of cell cycle-related genes. Although no statistical differences in the expression of *Cdkn1b* (p27), *Cdkn2b* (p15), or *Cdk6* were detected (data not shown), *Ccnd1* (cyclin D1) expression was significantly increased in Meis1-deficient LSK cells, when compared to that in sham-treated control LSK cells (**Figure 5A**). Taken together, these findings indicate that Meis1 regulates genes involved in the self-renewal and cell cycle of HSC/HPCs.

## Discussion

Despite the overwhelming evidence that Meis1 is involved in leukemogenesis, its normal physiological functions remain unclear. By using an inducible conditional knockout approach in adult mice, we have demonstrated here that *Meis1* deletion results in a loss of HSC/HPCs, subsequently causing multi-lineage BM failure. Specific deletion of *Meis1* within the hematopoietic system demonstrates a cell-autonomous requirement for Meis1 in maintaining the adult HSC/HPCs. In the HSC compartment, loss of *Meis1* enhanced cell cycle entry, with almost complete loss of the most quiescent SP cells, although cell survival was not affected. Together, our data provide evidence that Meis1 functions in maintaining long-term hematopoiesis via regulating the cell cycle status of HSC/HPCs.

Quiescence of stem cells has been postulated to prevent their exhaustion and is tightly linked to maintenance of the long-term self-renewal capacity of tissue stem cells, including HSCs [28]. Several transcriptional regulators have been shown to play key roles in this process. In the absence of Meis1, cells in the HSC compartment appear to exit from their quiescent state, as evidenced by the loss of the SP fraction from LSK cells, and undergo cell-cycle entry without an increase in this compartment, suggesting that Meis1 regulates self-renewal capacity of HSCs but not their simple expansion or differentiation. This notion was further supported by our *in vitro* colony formation assays using CD34^−^ LSK cells, in which *Meis1* deficiency significantly reduced the numbers and size of colonies but did not affect their potential to differentiate into various types of colonies, with an exceptional lack of mixed type colonies. Therefore, we postulate that Meis1 functions in replication of HSC/HPCs rather than in their differentiation. In this regard, a similar cell context-dependent role in cell cycle control was observed in MLL1-deficiency, in which the loss of MLL1, a potential upstream regulator of *Meis1* in the hematopoietic cell compartment, induced cell cycle progression in the HSC compartment whereas it reduced the proliferation of progenitor populations [30]. Thus, it is possible that Meis1 is a core molecule downstream of MLL1 that regulates the self-renewal capacity of HSCs via regulating their cell cycle status.

Another important protein that is associated with Meis1 in the HSC compartment and is possibly involved in cell cycle regulation is Pbx1, a directly interacting Meis1 partner in DNA binding [15]. Pbx1 is the most highly expressed Pbx family member in HSCs and is a critical regulator of self-renewal of adult HSCs [5]. Pbx1 maintains quiescence, as its absence results in the loss of LSK cells and increased cell cycle entry [5], similar to the defects we observed in LSK cells upon *Meis1* deletion. Our gene expression data also support the potential involvement of Pbx1-Meis1 heterodimers in HSC functions because there is overlap between the genes involved in HSC maintenance that are affected by both Meis1 and Pbx1 deficiencies, including *Msi2*, *Smad7* and *Hmga2*. Although there are some differences in the expression of cell cycle regulators, it is reasonable to assume that Meis1 functions in part by maintaining the cell cycle as well as self-renewal of the HSC compartment via Pbx1. Indeed we found that the transcriptional regulatory regions of most of these genes, such as *Smad7*, *Msi2* and *Hmga2*, contain Meis1-binding sequences in close proximity to Pbx-binding sites (data not shown). The accumulation of Meis1 as well as Pbx1 on *Smad7* and *Msi2* was identical to those in hematopoietic stem and progenitor cells demonstrated by Chip-seq analysis in a previous study [31].

Among the genes potentially regulated by both Meis1 and Pbx1 in HSCs, *Msi2*, is of particular interest [32], [33], since it encodes Musashi2, an RNA-binding protein preferentially expressed in adult HSCs. *Msi2* was also reported to be up-regulated in leukemic progenitors transduced with Vp16-Meis1, a fusion of the Vp16 activation domain and Meis1 [29], consistent with the observed reduction in its expression in *Meis1*-deficient LSK cells. Similar to the reported function of the *Drosophila* ortholog of Msi2 as a critical regulator of asymmetric cell division of the sensory organ precursor [34], overexpression of *Msi2* in cultured LSK cells increased asymmetric cell division [32], a phenomenon that has long been thought to be involved in the regulation of HSC self-renewal and differentiation [35]. Moreover, *Msi2*-deficiency in mice caused a severe decrease in HSCs [36], particularly in the CD34^+^ LSK compartment with mild effects in the CD34^−^ LSK compartment, which is similar to the observed phenotype of *Meis1*-deficient LSK cells. Cell cycle entry as well as *Ccnd1* expression was reduced in *Msi2*-deficient LSK cells [32], suggesting that *Meis1*-deficiency may perturb other factors in addition to Msi2. Since *Msi2* loss affects HSC self-renewal more severely under stress conditions [36], Mx1-Cre/interferon-mediated *Meis1*-deletion may alter the balance of asymmetric division, symmetric renewal/commitment in the HSC compartment, thereby promoting their differentiation rather than maintaining their self-renewal (**Figure 5B**). As Meis-Pbx-Hox orthologs in *C. elegans* have been shown to participate in cell fate determination in association with asymmetric cell division [37], the important role of Meis1 in asymmetric/symmetric division of cells in the HSC compartment is also highlighted by data showing that NUP98-HOXA9, an oncogenic partner of Meis1 in human myeloid leukemia [16], [38], promotes symmetric self-renewal of hematopoietic precursors [39].

One unexpected observation is that *Hif1a* expression in LSK cells was not significantly affected by *Meis1* deletion, although *Hif1a* contains an evolutionarily conserved Meis1-binding site in the first intron through which Meis1 transactivates its expression [11]. We have found a Meis1 binding peak 27 kb upstream of *Hif1a* exon 1 by Chip-seq analyses and a reduction in its expression by conditional deletion of *Meis1* in AML cells (T. N., unpublished data), although another study did not show such a binding peak within the *Hif1a* locus [37]. During submission of the present paper, two other groups described the HSC phenotype of Meis1-deficient mice [40], [41]. Although the HSC phenotypes they reported are almost identical with those described in the current study, the role of Meis1 in *Hif1a* expression was somewhat different among these three reports. The discrepancy regarding Meis1-mediated regulation of *Hif1a* expression in HSCs between our findings in the present study and those of both Unnisa *et al*. and Kocabas *et al*. could be due to differences in the cell type (LSK versus CD34^−^ LSK or Lin^−^ BM cells) or methods used for *Meis1* deletion, interferon-mediated Mx1-Cre versus tamoxifen-mediated CreER, which may affect the relative contribution of Meis1 in regulating *Hif1a* expression. Thus, it is likely that Meis1-mediated *Hif1a* expression is strictly cell context- and/or differentiation stage-dependent. However, we cannot exclude a potential contribution of *Hif1a* in HSCs as one of the downstream targets of Meis1.

In conclusion, we demonstrate that Meis1 is required for the maintenance of adult hematopoiesis in the BM. Meis1 has also been reported to maintain the undifferentiated state and expansion of retinal progenitor cells [42], [43], and the loss of *Meis1* causes premature differentiation of these cells. Thus, further investigation of the molecular mechanisms underlying the functions of Meis1 in HSCs, such as its potential functional cooperation with Pbx1 and/or Hox, as well as its involvement in asymmetric/symmetric cell division of HSCs, should facilitate our understanding of transcriptional networks regulating the maintenance of stem cells as well as their neoplastic counterparts.

## Materials and Methods

### Ethics statement

All animal experiments were carried out under the ethical guidance of Tokyo University of Science, and protocols were reviewed and approved by the Tokyo University of Science Animal Care and Use Committee.

### Mice and gene targeting

The *Meis1* targeting vector was assembled in a pKSTKLoxPNeo plasmid containing appropriate *loxP* sites, a *loxP*–flanked *PGK* promoter-driven *neo* gene, and the HSV thymidine kinase gene. The homologous regions of the final vector consisted of a 0.9 kb genomic fragment immediately upstream of the *lox*P-flanked 0.5 kb fragment containing exon 8 of the *Meis1* gene and a 6.5 kb DNA fragment immediately downstream of the gene. To establish mice carrying the *Meis1* floxed allele, the linearized targeting vector was electroporated into E14 ES cells, and drug-resistant colonies were screened for homologous recombination. Targeted clones were injected into C57BL/6 blastocysts and the resultant chimeric mice were bred to produce progeny capable of germ line transmission of the mutated allele. To remove the *loxP*-flanked neomycin-resistant gene cassette, mice harboring a targeted *Meis1* allele with the neomycin-resistant gene (*Meis1* ^neo/+^) were crossed with *EIIa*-Cre transgenic mice [44] and the resultant *Meis1^fln/^* ^+^ *EIIa*-Cre (mosaic) mice were crossed with C57BL/6 mice to establish *Meis1^fl/^* ^+^ mice and also *Meis1* ^Δ*/+*^ mice. *Meis1^fl/^* ^+^ mice were backcrossed at least eight times onto the C57BL/6 background and then were crossed with *Mx1-Cre* [23]. *Mx1-Cre* ^+^ *Meis1^fl/fl^* and *Meis1^fl/fl^* mice (controls) at the age of four to eight weeks were injected *i.p.* with 1.5 µg/weight (g) of poly(I:C) (GE Healthcare Bioscience) four times at one-day intervals to activate the interferon gene.

### Flow cytometry and cell sorting

Single cell suspensions from the indicated organs were stained with a combination of FITC-, PE-, -APC, -APC-Cy7 and biotin-conjugated antibodies, followed by streptavidin-PerCP-Cy5.5 or streptavidin-PerCP-Cy7 (eBioscience, San Diego, CA). Conjugated and unconjugated antibodies specific for the following antigens were purchased from BD Biosciences (San Jose, CA) and eBioscience: TCRαβ (H57-597), NK1.1 (PK136), CD3ε (145-2C11), CD11c (N418), CD135/flt3 (A2F10), B220 (RA3-6B2), c-kit (2B8), CD4 (L3T4), CD8a (53-6.72), CD11b (M1/70), CD19 (1D3), TER119, Gr-1 (RB6-8C59), FcγII/III (2.4G2), Sca-1 (E13-161.7), IL-7Rα (A7R34), CD41 (MWReg30), CD43 (S7), CD34 (RAM34), CD45.1 (A20), and CD45.2 (104). For lineage markers, CD11b, Gr-1, TER119, B220, and CD3 were used. SP population was analyzed as previously described [27]. Data were collected on a FACSCalibur or a FACSCanto™II flow cytometers (BD Bioscience) and analyzed using FlowJo software (TreeStar, Ashland, OR). A MoFlo (DAKO) or a FACSArea™II (BD Bioscience) was used for cell sorting.

### Bone marrow reconstitution assays

Sorted CD34^−^ LSK cells (50 cells) from *Mx1-Cre* ^+^ *Meis1^fl/fl^* or control *Meis1^fl/fl^* mice (CD45.2) were mixed with sorted CD34^−^ LSK cells (50 cells) from CD45.1/CD45.2 competitor mice, and then intravenously injected into lethally irradiated CD45.1 recipients with 2×10^5^ CD45.1 wild-type bone-marrow (BM) mononuclear cells (MNCs). Three months after the transplantation, one half of each recipient group was treated with poly(I:C), and peripheral blood cells were collected monthly to examine the percentage of donor-derived cells based on CD45.2 expression. Three months after the poly(I:C) treatment, the recipient mice were terminated to examine the donor-derived HSC and their contribution to different lineages. In addition, BM MNCs (2×10^5^) from each recipient group were further used for secondary transplantation into lethally irradiated CD45.1 recipients.

### Methylcellulose assays

Sorted CD34^−^ LSK cells (100 cells) from *Mx1-Cre* ^+^ *Meis1^fl/fl^* or *Meis1^fl/fl^* mice pretreated with poly(I:C) were plated in methylcellulose medium (Stem Cell Technologies) supplemented with mixtures of cytokines. The culture dishes were incubated at 37°C in a 5% CO_2_ humidified atmosphere, and colony numbers were counted at day 14. Colonies (>1 mm in diameter) were recovered and subjected to May-Grünwald Giemsa staining for morphological examination.

### Cell cycle and apoptosis analyses

For bromodeoxyuridine (BrdU) incorporation analysis, BrdU (1 mg per mouse: BD Pharmingen) was injected intraperitoneally. At one h post-injection, LSK cells were collected from the BM, fixed, and stained with 7-AAD and anti-BrdU antibody using a FITC-BrdU Flow kit (BD Pharmingen), according to the manufacturer's protocol. To assess apoptosis, cells stained for stem cell markers were further incubated with Annexin V and propidium iodide (PI).

### Microarray analysis

RNA was isolated using the Qiagen RNeasy micro kit (Qiagen) from LSK cells from poly(I:C)-treated *Mx1-Cre* ^+^ *Meis1^fl/fl^* and sham-treated *Meis1^fl/fl^* mice (pools of 6 mice; 2 pools per genotype). Ten ng of total RNA was amplified using the WT-Ovation™ Pico RNA Amplification system (NuGEN Technologies, Inc.) and labeled using the Genomic Enzymatic Labeling Kit (Aligent Technologies). Labeled probes were hybridized on 4×44 K Whole Mouse Genome Oligo Microarrays (Aligent) and scanned with an Aligent Microarray Scanner. Microarray signals and background information were retrieved using Feature Extraction Software (v.9.5.3.1). All data analyses were performed using the GeneSpring software GX11.0.2 (Agilent). Genes with a raw *P*-value<0.01 and a fold-change greater than 2-fold were defined as differentially expressed. Array data are available at Gene Expression Omnibus (GEO accession number: GSE38336).

### RT-PCR analysis

Using the Qiagen RNeasy micro kit (Qiagen), total RNA was isolated from LSK cells from poly(I:C)-treated *Mx1-Cre Meis1* ^fl/fl^ and sham-treated *Meis1* ^fl/fl^ mice (pools of six mice; two pools per genotype). Total RNAs were reverse transcribed using a SuperScript VILO cDNA Synthesis System (Invitrogen, Carlsbad, CA). Real-time RT-PCR was performed with Power SYBR Green PCR Master Mix (Applied Biosystems, Foster City, CA) and ABI 7500 Fast thermocycler (Applied Biosystems), according to the manufacturer's protocol. Amplification of *β-actin* was used to normalize for sample RNA content. Specificity of products was confirmed by melting curve analysis, assessing band size in 2% agarose gels, and DNA sequencing. The primer sequences used for RT-PCR and qPCR are listed in **Table S2**.

### Statistical analysis

Statistical significance was calculated using an unpaired two-tailed Student's *t*-test. Data were considered statistically significant when p values were less than 0.05.

## Supporting Information

Figure S1 **Preferential expression of** ***Meis1*** **in HSCs.** Expression of *Meis1*, *Meis2*, *Meis3*, and *GAPDH* genes was examined by semiquantitative RT-PCR analysis. cDNAs were prepared from CD34^−^ LSK cells, CD34^+^ LSK cells, lineage marker^−^ cells, Gr-1^+^ neutrophils, Mac-1^+^ macrophages, TER119^+^ erythroblasts, and B220^+^ B-lineage cells from BM; from B220^+^ B cells and CD3^+^ T cells from the spleen; and, from CD4^−^CD8^−^ (DN), CD4^+^CD8^+^ (DP), CD4^+^CD8^−^ (CD4 SP), and CD4^−^CD8^+^ (CD8 SP) T-lineage cells in the thymus of adult wild-type mice.
(TIF)

Figure S2 **Generation of conditional and deleted** ***Meis1*** **alleles.** (**A**) A diagram depicting exon 8 of the *Meis1* locus and the targeting strategy used to generate two targeted versions of the *Meis1* allele (floxed and deleted alleles). *LoxP* sites (arrowheads) were inserted into intronic sites flanking exon 8 of the *Meis1* gene. Correct targeting was verified by Southern blot analysis of *Hind*III-digested DNA with the indicated probe (filled rectangle). The lengths of the respective *Hind*III fragments are shown in kb. PCR primers for verifying the Cre-mediated deletion of the *loxP*-flanked fragment are indicated by arrows. Neo, neomycin-resistant gene; tk, thymidine kinase gene; H, *Hind* III. (**B**) Southern blot analysis of germline transmission of the mutated *Meis1* alleles. Tail DNA from the indicated mice was digested with *Hind* III and hybridized with the probe indicated in (**A**). (**C**) Confirmation of *Meis1* deletion in *Mx1-Cre* ^+^ mice. DNAs from sorted LSK cells from *Mx1-Cre* ^+^ *Meis1* ^fl/fl^ mice that were either treated (+) or untreated (−) with poly(I:C) were subjected to PCR analysis using primer pairs shown in (**A**). DNAs from *Meis1* ^Δ/Δ^ mice were used as controls.
(TIF)

Figure S3 **PCR genotyping of hematopoietic cells from** ***Mx1-Cre^+^ Meis1*** **^fl/fl^ and control** ***Meis1*** **^fl/fl^ mice three weeks after poly(I:C) treatment.** Efficient excision of floxed Meis1 alleles was observed in sorted Gr-1^+^ CD11b^+^ mature granulocytes.
(TIF)

Figure S4 **Loss of Meis1 abrogates megakaryocyte lineage differentiation in the bone marrow.** Representative flow cytometric profiles of megakaryocytic-lineage cell populations from *Mx1-Cre* ^+^ *Meis1* ^fl/fl^ and control *Meis1* ^fl/fl^ mice three weeks after poly(I:C) treatment. Gates used to identify megakaryocytic-lineage cell populations (Lin^−^) are outlined, and rightward arrows indicate their relationship to subsequent plots showing the megakaryocyte precursors (cKit^+^ CD41^+^) and mature megakaryocytes (cKit^−^ CD41^+^). Numbers within the analysis gates indicate percentage of gated cells in total BM mononuclear cells. Bar graphs on the right represent absolute numbers of the indicated cell populations per two femurs in poly(I:C)–treated *Mx1-Cre* ^+^ *Meis1* ^fl/fl^ (solid bars) and control *Meis1* ^fl/fl^ (open bars) mice (mean and SD; n = 4). **p<0.05.
(TIF)

Figure S5 **Loss of Meis1 severely impairs T cell development in the thymus.** (**A**) Representative flow cytometric profiles of T cell progenitor populations in the thymus from *Mx1-Cre^+^ Meis1* ^fl/fl^ and control *Meis1* ^fl/fl^ mice three weeks after poly(I:C) treatment. Numbers in each quadrant indicate percentage of gated cells in total thymocytes. (**B**) Absolute numbers of CD4^−^CD8^−^ (DN), CD4^+^CD8^+^ (DP), CD4^+^CD8^−^ (CD4 SP), and CD4^−^CD8^+^ (CD8 SP) cell populations in poly(I:C)–treated *Mx1-Cre^+^ Meis1* ^fl/fl^ (solid bars) and control *Meis1* ^fl/fl^ (open bars) mice (mean and SD; n = 4). *p<0.05 and **p<0.01.
(TIF)

Table S1 **The list of genes differentially expressed between Meis1-deficient and –sufficient LSK cells.**
(XLSX)

Table S2 **The list of primers for qPCR.**
(XLSX)
