# Supplementary figures and images for: Homeodomain Transcription Factor Meis1 Is a Critical Regulator of Adult Bone Marrow Hematopoiesis
Source: PLoS One. 2014 Feb 3;9(2):e87646. doi: 10.1371/journal.pone.0087646 (PMC3911998; doi:10.1371/journal.pone.0087646)

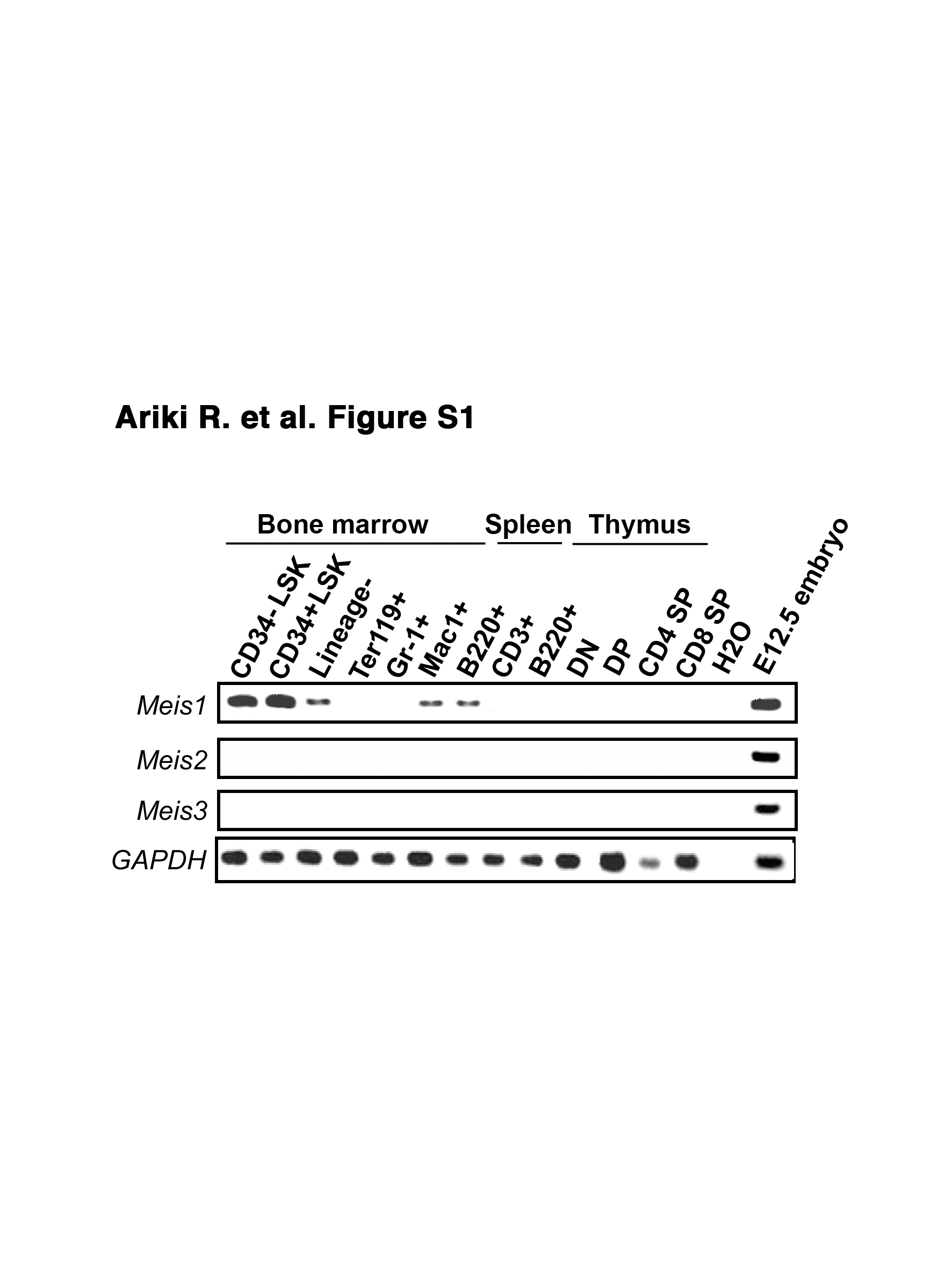

Supplement: Figure S1 — Preferential expression of Meis1 in HSCs. Expression of Meis1, Meis2, Meis3, and GAPDH genes was examined by semiquantitative RT-PCR analysis. cDNAs were prepared from CD34− LSK cells, CD34+ LSK cells, lineage marker− cells, Gr-1+ neutrophils, Mac-1+ macrophages, TER119+ erythroblasts, and B220+ B-lineage cells from BM; from B220+ B cells and CD3+ T cells from the spleen; and, from CD4−CD8− (DN), CD4+CD8+ (DP), CD4+CD8− (CD4 SP), and CD4−CD8+ (CD8 SP) T-lineage cells in the thymus of adult wild-type mice. (TIF) [file pone.0087646.s001.tif]

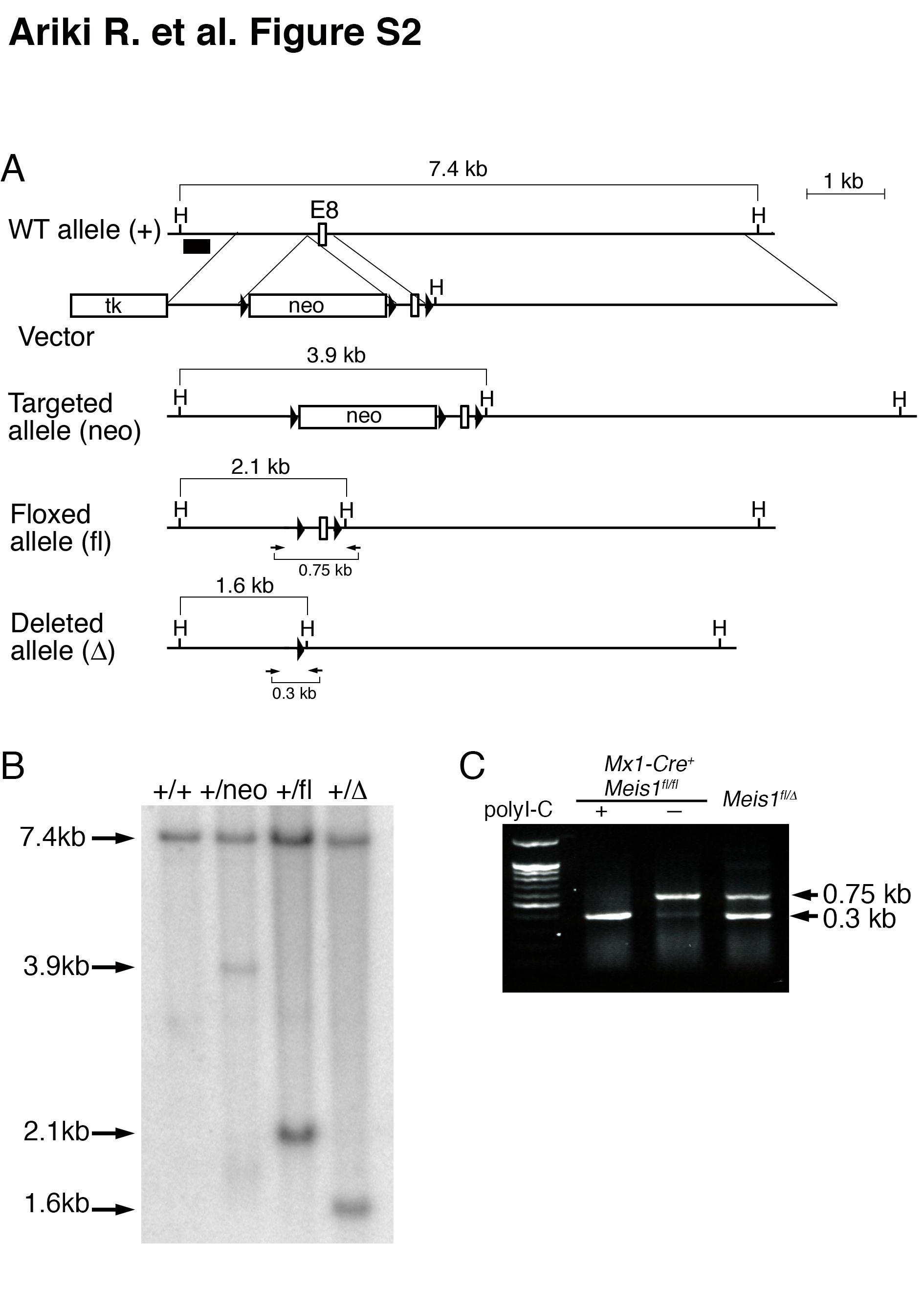

Supplement: Figure S2 — Generation of conditional and deleted Meis1 alleles. (A) A diagram depicting exon 8 of the Meis1 locus and the targeting strategy used to generate two targeted versions of the Meis1 allele (floxed and deleted alleles). LoxP sites (arrowheads) were inserted into intronic sites flanking exon 8 of the Meis1 gene. Correct targeting was verified by Southern blot analysis of HindIII-digested DNA with the indicated probe (filled rectangle). The lengths of the respective HindIII fragments are shown in kb. PCR primers for verifying the Cre-mediated deletion of the loxP-flanked fragment are indicated by arrows. Neo, neomycin-resistant gene; tk, thymidine kinase gene; H, Hind III. (B) Southern blot analysis of germline transmission of the mutated Meis1 alleles. Tail DNA from the indicated mice was digested with Hind III and hybridized with the probe indicated in (A). (C) Confirmation of Meis1 deletion in Mx1-Cre + mice. DNAs from sorted LSK cells from Mx1-Cre + Meis1 fl/fl mice that were either treated (+) or untreated (−) with poly(I:C) were subjected to PCR analysis using primer pairs shown in (A). DNAs from Meis1 Δ/Δ mice were used as controls. (TIF) [file pone.0087646.s002.tif]

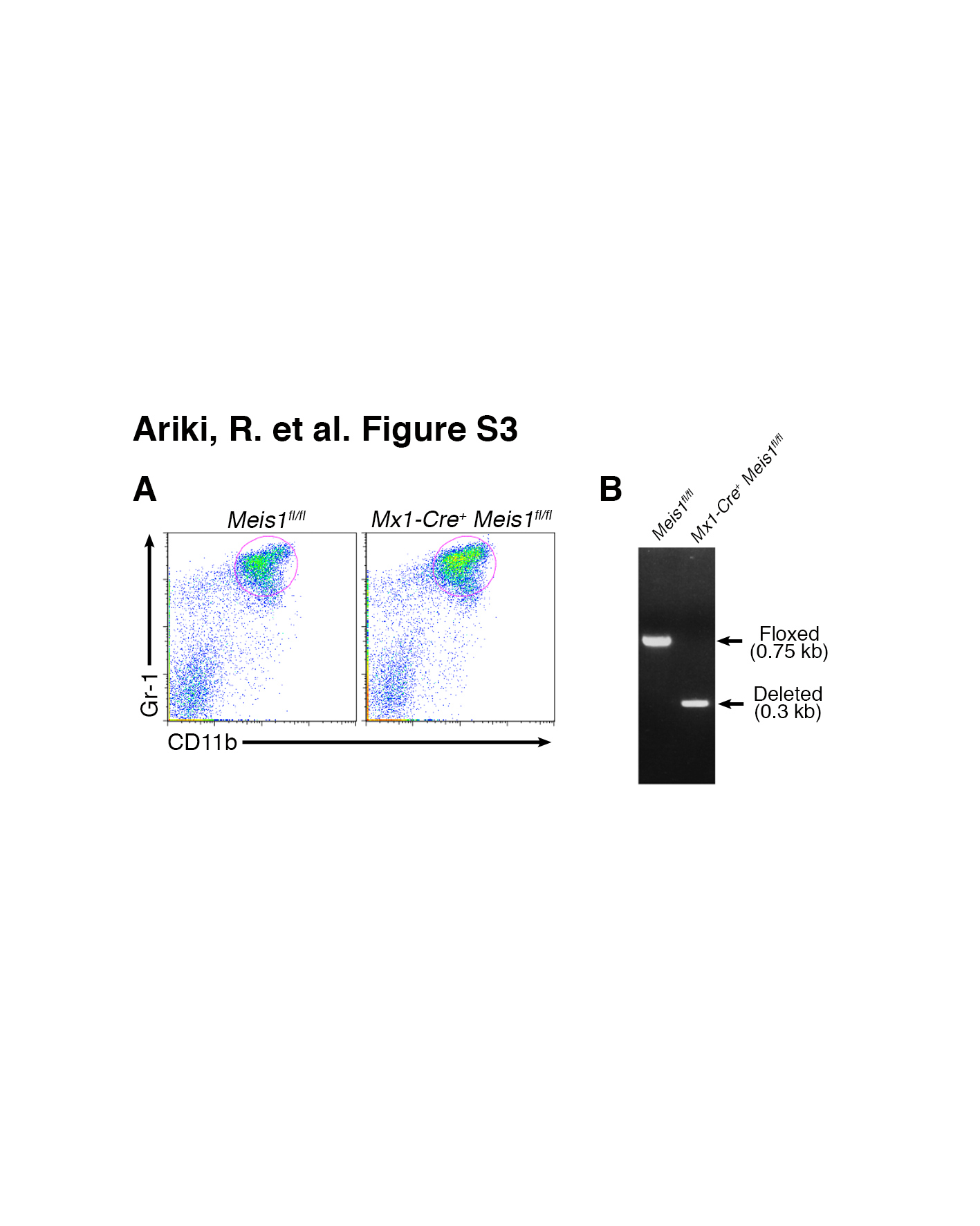

Supplement: Figure S3 — PCR genotyping of hematopoietic cells from Mx1-Cre+ Meis1 fl/fl and control Meis1 fl/fl mice three weeks after poly(I:C) treatment. Efficient excision of floxed Meis1 alleles was observed in sorted Gr-1+ CD11b+ mature granulocytes. (TIF) [file pone.0087646.s003.tif]

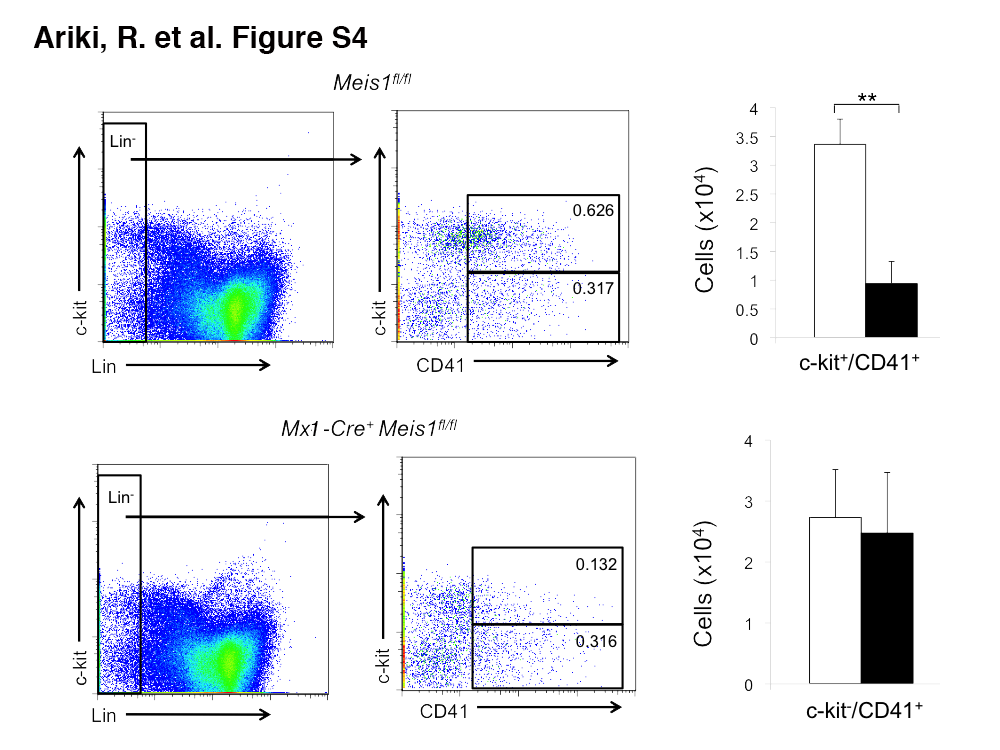

Supplement: Figure S4 — Loss of Meis1 abrogates megakaryocyte lineage differentiation in the bone marrow. Representative flow cytometric profiles of megakaryocytic-lineage cell populations from Mx1-Cre + Meis1 fl/fl and control Meis1 fl/fl mice three weeks after poly(I:C) treatment. Gates used to identify megakaryocytic-lineage cell populations (Lin−) are outlined, and rightward arrows indicate their relationship to subsequent plots showing the megakaryocyte precursors (cKit+ CD41+) and mature megakaryocytes (cKit− CD41+). Numbers within the analysis gates indicate percentage of gated cells in total BM mononuclear cells. Bar graphs on the right represent absolute numbers of the indicated cell populations per two femurs in poly(I:C)–treated Mx1-Cre + Meis1 fl/fl (solid bars) and control Meis1 fl/fl (open bars) mice (mean and SD; n = 4). **p<0.05. (TIF) [file pone.0087646.s004.tif]

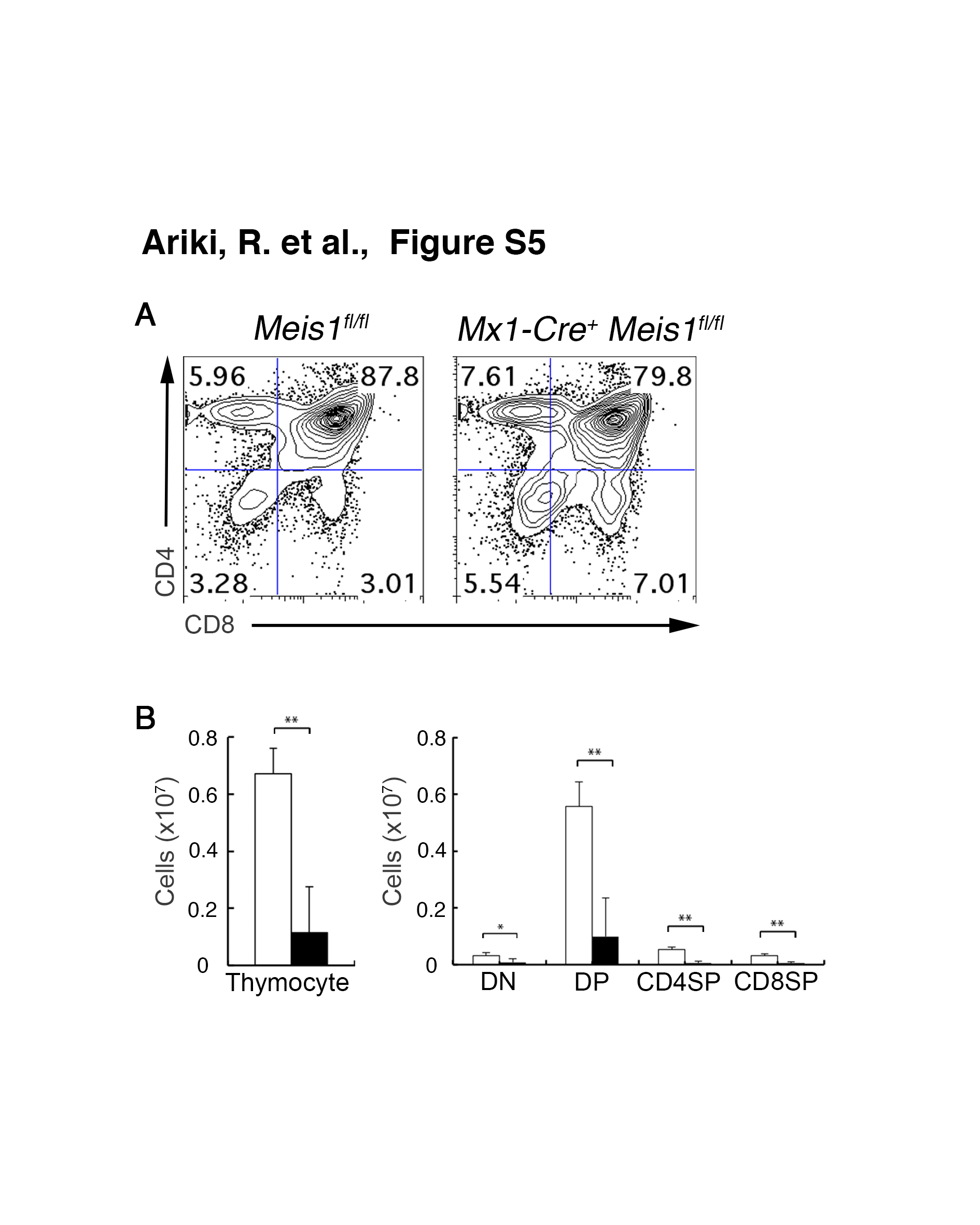

Supplement: Figure S5 — Loss of Meis1 severely impairs T cell development in the thymus. (A) Representative flow cytometric profiles of T cell progenitor populations in the thymus from Mx1-Cre+ Meis1 fl/fl and control Meis1 fl/fl mice three weeks after poly(I:C) treatment. Numbers in each quadrant indicate percentage of gated cells in total thymocytes. (B) Absolute numbers of CD4−CD8− (DN), CD4+CD8+ (DP), CD4+CD8− (CD4 SP), and CD4−CD8+ (CD8 SP) cell populations in poly(I:C)–treated Mx1-Cre+ Meis1 fl/fl (solid bars) and control Meis1 fl/fl (open bars) mice (mean and SD; n = 4). *p<0.05 and **p<0.01. (TIF) [file pone.0087646.s005.tif]
